# Air pollution and impulsive choice in aging: evidence from delay discounting

**DOI:** 10.1007/s11357-025-01906-0

**Published:** 2025-09-24

**Authors:** Maya R. Kilcullen, Jamie-Nicole Luistro, Melanie Kos, Jeremy Mennis, David V. Smith, Ingrid R. Olson

**Affiliations:** 1 Department of Psychology and Neuroscience, Temple University, 1701 N. 13, Philadelphia, PA USA; 2 Department of Geography, Environment, and Urban Studies, Temple University, Philadelphia, PA USA

**Keywords:** Impulsivity, Reward, Environment, Pollution, Aging

## Abstract

Heightened air pollution exposure is associated with an increased risk for developing neurodegenerative disorders such as Alzheimer’s disease and Parkinson’s disease, yet it is unclear how pollution impacts the aging brain more broadly. Rodent research has shown that higher air pollution exposure is associated with increased impulsive behavior, operationalized as a preference for immediate reward in delay discounting tasks. We examined this relationship in middle-aged and older humans by analyzing associations between residential pollution exposure and time-based reward preferences. One hundred three (103) adults aged 40–80 completed a delay discounting task. We estimated long-term residential exposure to fine particulate matter (PM_2.5_), a prominent air pollutant, using participant address information and satellite pollution data. We found that higher residential PM_2.5_ exposure was significantly associated with a higher preference for more immediate rewards, even after controlling for demographic factors including income and education. A preference for immediate rewards has been independently associated with a higher risk of addictive behaviors, including substance abuse and gambling disorders, indicating that PM_2.5_ exposure may increase the emergence of these conditions. We discuss these results in detail along with potential underlying biological mechanisms, implications on human behavior, and future research directions.

**Supplementary information:**

The online version contains supplementary material available at 10.1007/s11357-025-01906-0.

## Introduction

Air pollution in the USA is a growing public health issue, with 156.1 million people—approximately 46% of the country’s population—living in counties with substandard air quality, according to the American Lung Association [1]. While discourse around air pollution and human health has centered on respiratory concerns like asthma or lung cancer, there is emerging evidence that poor air quality has a cognitive burden [2].

Alzheimer’s-focused research has found that higher air pollution exposure is associated with faster cognitive decline and poorer executive functioning in individuals with the disease or those genetically predisposed to it (e.g., APOE-ε4 carriers) [3–5]. While this work is important, it leaves open a critical question: can air pollution also impair cognition in the broader aging population, beyond those already diagnosed with a neurodegenerative disease?

Rodent models suggest the answer may be yes. In healthy adult animals, chronic exposure to fine particulate matter (PM_2.5_, aerodynamic diameter < 2.5 μm), a common air pollutant often containing components of soot, smoke, and trace metals, has been shown to increase impulsivity across multiple paradigms. For example, exposed rodents were more likely to jump off a high ledge and do so at a faster rate during the Cliff Avoidance Reaction (CAR) test, a common impulsivity test in rodent research [6]. They also display steeper delay discounting, reflecting a greater preference for smaller, immediate rewards over larger, delayed ones— a behavioral marker often interpreted as increased impulsive choice [7, 8]. These behavioral changes are supported by evidence of pollution-induced inflammation in the striatum, a key brain region involved in reward processing and executive function [9, 10].

In humans, steeper discounting (i.e., stronger preference for immediate rewards) is frequently used as a behavioral index of impulsivity and has been associated with a range of maladaptive outcomes, including substance use disorders, gambling disorders, lower income attainment, and poorer psychiatric health [11, 12]. Despite the significant consequences, the relationship between PM_2.5_ exposure and delay discounting has not yet been examined in humans.

Understanding this potential link is especially important for middle-aged and older adults, who frequently face important financial decisions about retirement, healthcare, insurance, and long-term planning. Although some studies suggest discounting decreases with age, individual variability persists throughout the lifespan, and even subtle changes in cognitive abilities could carry consequences for older adults [13].

Building on rodent findings, we investigated whether long-term exposure to PM_2.5_ is associated with higher impulsive choice in a community sample of middle-aged and older adults. Specifically, we examined whether higher PM_2.5_ exposure would predict a greater preference for smaller, immediate rewards, as measured by performance on the convex time budget (CTB) task, a validated delay discounting paradigm [14].

## Materials and methods

### Participants

Data collection was part of an on-going 5-year study (2021–2026) funded by the National Institute on Aging (NIA) [15]. The parent study included individuals as young as 21 years of age. Study eligibility was first assessed through online questionnaires collecting current address, demographic, and health information. Participants who self-reported any history of severe psychiatric disorders (e.g., schizophrenia, bipolar disorder), major neurological conditions (e.g., epilepsy, brain tumor, large-vessel stroke, major head trauma, dementia), and current systemic or metabolic illnesses (e.g., B12 deficiency, renal failure, cancer) were deemed ineligible for this study. Individuals with severe sensory or motor impairments that would prevent completion of study measures were also excluded. For MRI-based components of the parent study, participants also had to meet standard MRI safety requirements (e.g., no metallic implants, no history of working with metal fragments, no claustrophobia).

Eligible participants were then invited to Temple University to complete a variety of behavioral tasks, including the convex time budget (CTB) task. For the present analyses, we took a data-driven approach and included all participants who completed the full battery of in-person tests and met our age requirements.

We limited the analytic sample to participants over the age of 40 for two primary reasons. First, our focus is on aging and its early precursors, particularly the emergence of subtle cognitive changes that would precede any clinical diagnosis. While many studies of older adults use age 55 as a lower threshold, research suggests that aspects of cognitive aging may emerge much earlier [16]. Including participants beginning at age 40 allowed us to capture potential prodromal symptoms of cognitive change. Second, our pollution exposure estimates are based on current residential addresses (see Air Pollution Assessment). National census data demonstrate that younger adults tend to change residences more frequently than older adults; restricting the sample to participants over 40 increases the likelihood of stable long-term exposure estimates [17]. The final analytic sample consisted of 103 participants (59 female, 44 male) from the Philadelphia, Pennsylvania, metropolitan area, ranging in age from 40 to 80 years (*M*_age_ = 59.1, SD = 10.8). Demographics are reported in Table 1. All participants provided consent in accordance with the Institutional Review Board at Temple University and were reimbursed for their time.

**Table 1:** Demographic information

Characteristic	Mean/*N*	SD/%
Age (years)	59.1	10.8
Race		
American Indian	1	1.0%
Asian	2	1.9%
Black	24	23.3%
White	74	71.8%
Two or more races/race not described	2	1.9%
Ethnicity		
Hispanic or Latino	4	3.9%
Not Hispanic or Latino	91	88.3%
Ethnicity not described	7	6.8%
Prefer not to respond	3	2.9%
Gender		
Male	44	42.7%
Female	59	57.3%
Household income		
$0–$25,000	30	29.1%
$26,000–$50,000	22	21.4%
$51,000–$75,000	12	11.7%
$76,000–$100,000	17	16.5%
$101,000–$150,000	11	10.7%
$151,000–$200,000	10	9.7%
$201,000 or greater	1	1.0%
Education level		
Less than high school	1	1.0%
High school	11	10.7%
Trade qualification or certificate	4	3.9%
GED	6	5.8%
Some university	19	18.4%
Bachelor’s degree	24	23.3%
Postgraduate degree	38	36.9%

Participants were allowed to select more than one ethnicity

### Measures

#### Convex time budget (CTB) task

**Fig. 1:**
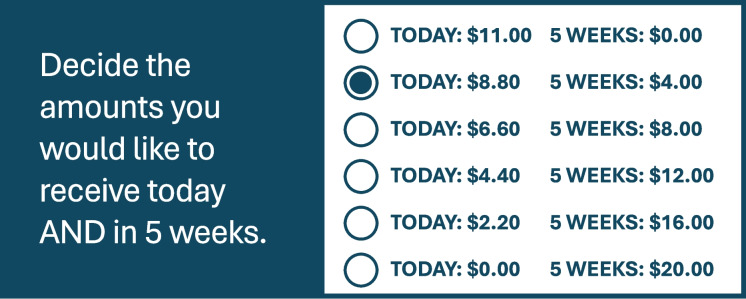
Sample convex time budget (CTB) question

The convex time budget (CTB) task is a validated delay discounting task that assesses participants’ time-based monetary reward preferences [14] (Fig. 1). Participants were instructed to choose their preferred pairwise monetary reward, split across sooner and later time points. Each trial contained six pairs of monetary rewards, ranging from all money received at the sooner time point to all money received at the later time point. Total monetary reward increased as weight shifted toward the later time points. Trials were categorized into four delay ranges:

1. Today vs. 5 weeks,
2. Today vs. 9 weeks,
3. 5 weeks vs. 10 weeks,
4. 5 weeks vs. 14 weeks.

Participants completed six trials per delay range, for a total of twenty-four trials. Compared to many delay discounting measures in which participants are only presented with two options (all now or all later), the scaling framework of the CTB task offers a more nuanced assessment of discounting behavior, making it well-suited for the aims of this study.

Scoring for this study followed a simplified framework designed to capture relative preference for sooner versus later rewards. On each trial, responses were coded on a 1–6 scale, with “1” indicating allocation of the full reward to the sooner time point (all now) and “6” indicating allocation of the full reward to the later time point (all later). Scores 2–5 indicate a monetary split between the two time points, with higher scores reflecting a higher proportion of delayed reward. The maximum delayed reward was always $20, and the sooner reward varied across trials to manipulate the attractiveness of immediate choice. For example, in the Today vs. 9 weeks condition, the first trial presented an obvious tradeoff (e.g., $20 today vs. $20 in 9 weeks), while subsequent trials gradually increased the incentive to wait (e.g., $11 today vs. $20 in 9 weeks). An average CTB score across the 24 trials was calculated for each participant, with higher mean scores reflecting greater preference for delayed rewards. This scoring approach provides an interpretable, continuous measure of discounting behavior.

#### Air pollution exposure assessment

**Fig. 2:**
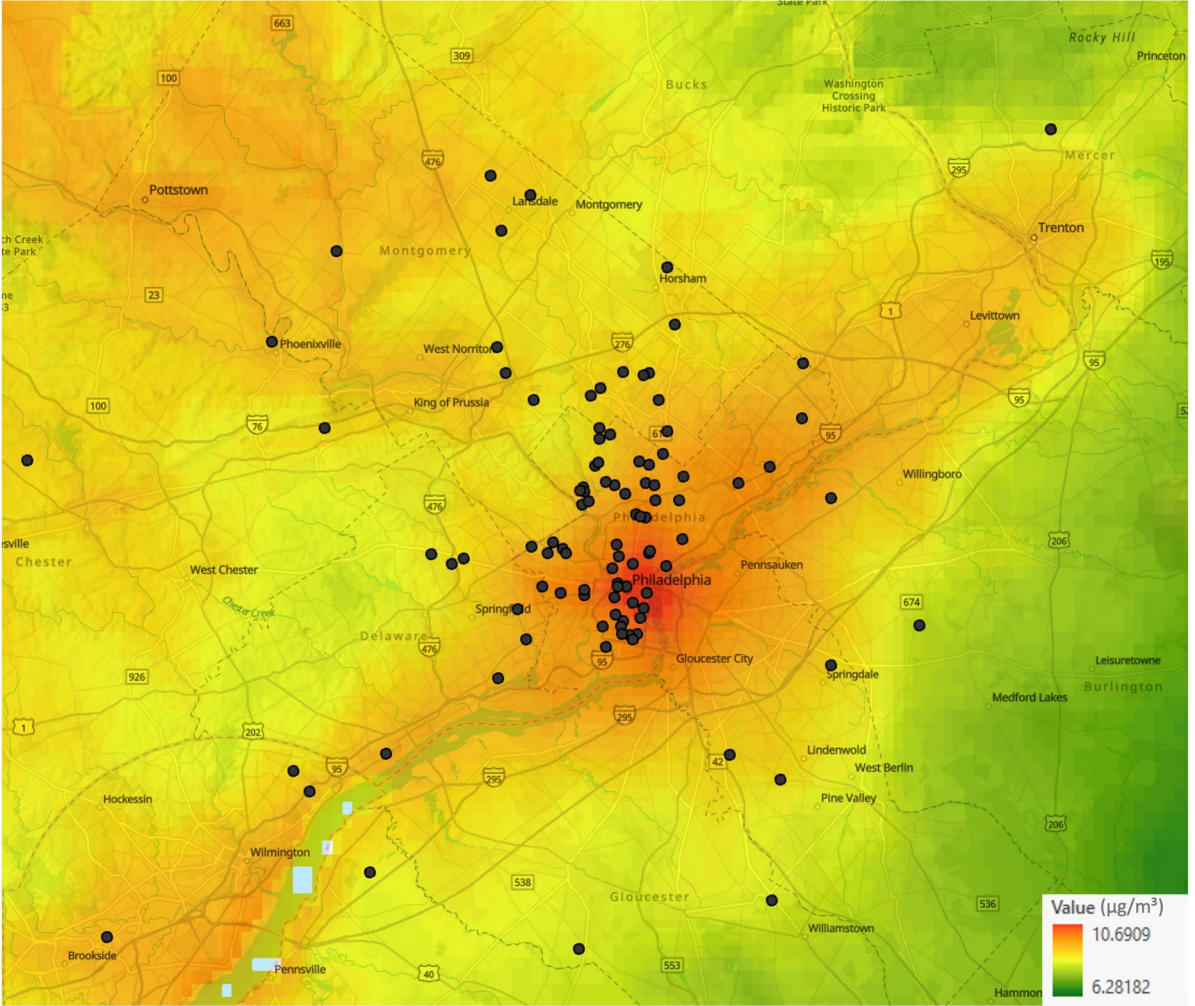
Map of PM_2.5_ concentration estimates (red = higher, green lower) for the greater Philadelphia, Pennsylvania region, 2012–2022. Each dot represents a study participant’s residential address location, randomly adjusted by 500 m to uphold confidentiality. Most participants were from eastern Pennsylvania; however, some came from the neighboring states of Delaware and New Jersey. Three participant addresses are outside of the image range and not depicted below

Residential addresses were collected from participants at the onset of study participation and geocoded using ArcGIS Pro v.3.3.0 software [18]. Annual average ground-level PM_2.5_ concentrations were represented using a geospatial North American PM_2.5_ concentration dataset derived from the integration of Aerosol Optical Depth (AOD) measurements from multiple satellite-based instruments from the National Aeronautics and Space Administration (NASA) with ground-based PM_2.5_ observations from the Environmental Protection Agency’s (EPA) Air Quality System (AQS) [19]. To account for seasonal fluctuations and transient pollution changes during the COVID-19 pandemic, we extracted an average PM_2.5_ concentration estimate for each geocoded residential address across the time window 2012–2022 (see Fig. 2).

### Statistical analysis

Statistical analyses and plotting were performed using R Studio, version 4.5.0. A linear multiple regression model was used to estimate the association between long-term residential PM_2.5_ exposure and average scores on the convex time budget (CTB) task, a measure of delay discounting. Age (in years), self-identified gender, annual household income, and education level (highest degree obtained) were included as covariates, based on prior research indicating their relevance to both delay discounting and air pollution exposure [20–27].

Participants selected their gender from four options (male, female, non-binary, other). However, among participants over age 40 (the analytic sample for this study), all identified as male or female; as such, only two gender categories are represented in Table 1 and in the regression models. Annual pre-tax household income was reported by choosing from a list of categorical ranges ($0–$25,000, $26,000–$50,000, $51,000–$75,000, $76,000–$100,000, $101,000–$150,000, $151,000–$200,000, greater than $201,000), which were coded on an ordinal scale for analysis. Personal income and participants’ self-rated social status were also collected but were excluded from the model due to high correlations with household income and education (see Supplementary Fig. 2).

Lower socioeconomic status (SES) has been associated with steeper delay discounting as well as a higher prevalence of related outcomes such as substance use and gambling disorders [20–25]. Prior studies have found that men typically exhibit greater impulsive choice behavior than women [22]. Although findings on age have been mixed, some evidence suggests a U-shaped association with delay discounting, with the lowest rates of discounting observed around midlife [27]. Controlling for these variables allowed us to more precisely estimate the unique contribution of PM_2.5_ exposure to delay discounting. Statistical significance was set at two-sided *p* < 0.05.

## Results

### Pollution estimation

Long-term average PM_2.5_ concentrations (2012–2022) in the sample ranged from 6.2 to 11.1 μg/m^3^. In 2024, the U.S. EPA revised the annual PM_2.5_ air quality standard from 12 to 9 μg/m^3^ [28]. Exposure levels in our sample spanned both sides of this revised threshold. The highest concentrations were observed in high-traffic, densely populated neighborhoods of Philadelphia (e.g., Center City) and in agriculture-dense rural zones in surrounding counties (see Fig. 2), consistent with our expectations and consistent with prior findings [29].

### Delay discounting distribution

CTB responses, scored on the 1–6 scale described in the Methods, yielded an average score of 3.88 (SD = 1.67) across participants. Higher scores reflect stronger preferences for delayed rewards. This average CTB score was used as the dependent variable in regression analyses.

### Regression results

**Fig. 3:**
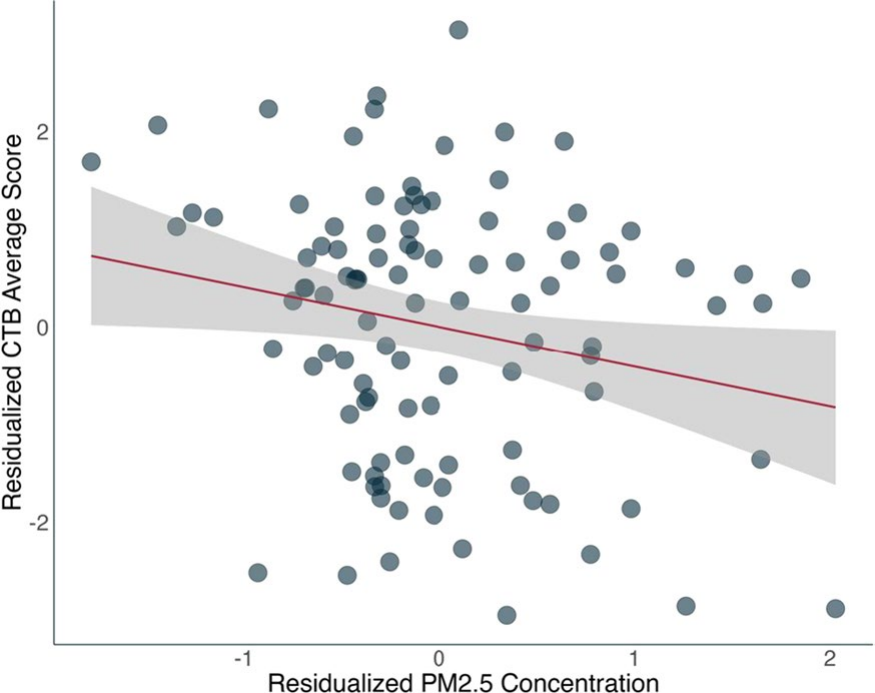
Higher exposure to PM_2.5_ is associated with more impulsive responses on the convex time budget (CTB) Task, after adjusting for age, gender, education, and household income. Both axes reflect *residualized values*, meaning we first used a linear model to statistically control for the effects of these covariates, then plotted the remaining (residual) variation in PM_2.5_ and CTB scores. This isolates the specific association between PM_2.5_ exposure and CTB performance, independent of the covariates. In our sample, raw PM_2.5_ exposure ranged from 6.2 to 11.1 μg/m^3^. For reference, the U.S. EPA’s current annual air quality standard is 9 μg/m^3^

A multiple linear regression was conducted to estimate the association between long-term PM_2.5_ exposure and delay discounting (CTB average scores) (Fig. 3). In an unadjusted model, PM_2.5_ exposure was a significant predictor of steeper discounting behavior (adjusted *β* = − 0.25, *p* = 0.011). PM_2.5_ alone explained 5.3% of the variance in the model (adj. *R*^2^ = 0.053). Even after controlling for age, gender, education, and household income, the relationship between PM_2.5_ and CTB scores remained significant (adjusted *β* = − 0.18, *p* = 0.034), suggesting that increased exposure to air pollution is associated with greater impulsivity. Among covariates, household income and education level were also statistically significant, with lower CTB scores (steeper discounting) associated with lower income and lower education, respectively (Table 2). The overall model was statistically significant, *F*(5, 97) = 11.28, *p* < 0.001, and accounted for approximately 33.5% of the variance in CTB scores (R^2^ = 0.335).

**Table 2:** Regression statistics

Predictor	*B* (Unstd.)	SE	*β* (Std.)	*t*	*p*
(Intercept)	5.67	1.94	–	2.93	0.004
PM_2.5_ concentration	− 0.41	0.19	− 0.18	− 2.15	0.034 *
Age	− 0.01	0.01	− 0.06	− 0.72	0.474
Gender (1 = female)	0.40	0.28	0.12	1.40	0.165
Education (scale)	0.36	0.09	0.36	3.97	< 0.001 ***
Household income (scale)	0.24	0.09	0.26	2.86	0.005 **

Model summary: *R*^2^ = 0.368, adjusted *R*^2^ = 0.335, F(5, 97) = 11.28, *p* < 0.001; residual SE = 1.361

Although the average CTB score was selected as the dependent variable for its simplicity and interpretability, we also applied a utility model to the raw CTB responses. This model assumes that participants make choices to maximize their subjective satisfaction (utility) and incorporates both the absolute dollar value and time delay of each option. This approach is consistent with the proposed framework by the original designers of the CTB task, using the estimated *delta* parameter as a more computationally grounded measure of delay discounting [30]. Average CTB scores and delta values were highly correlated (*r* = 0.94), and the association between PM_2.5_ exposure and discounting behavior remained significant when using delta as the dependent regressor (adjusted *β* = –0.27, *p* = 0.018). Full utility model results are reported in the Supplementary Materials (see Supplementary Fig. 1 and Supplementary Table 1).

## Discussion

To the authors’ knowledge, this study provides the first evidence in human adults linking exposure to fine particulate matter (PM_2.5_) with altered reward-based decision-making, specifically a greater preference for immediate over delayed rewards. Higher PM_2.5_ exposure was significantly associated with lower scores on the convex time budget (CTB) task, a behavioral measure of impulsive choice, even after controlling for age, gender, education, and household income. These findings build on rodent literature and offer a critical step toward understanding how environmental factors like PM_2.5_ may shape cognition and behavior in the broader aging population [6–10].

Although the precise mechanisms underlying the relationship between PM_2.5_ and decision-making are not fully established, chronic inflammation is widely considered a central pathway [31, 32]. The small size of particles allows inhaled PM_2.5_ to bypass biological protective mechanisms: particles can accumulate in the lungs and enter systemic circulation, and they may also reach the brain directly via the olfactory nerve, bypassing the blood–brain-barrier (BBB) [33]. Once inside the body, PM_2.5_, which is comprised of dust, soot, metals, and volatile compounds, can trigger sustained immune responses [34, 35]. Prolonged exposure to PM_2.5_ has been associated with increased levels of pro-inflammatory cytokines and reactive oxygen species (ROS), but the extent to which these changes affect reward-related neural systems remains an open question for future research [33].

The observed relationship between steeper delay discounting and higher PM_2.5_ exposure has several practical implications. Delay discounting has been linked to a wide range of maladaptive behaviors, including substance use and gambling [36–38]. Even in the absence of clinical disorders, individuals with steeper discounting rates tend to have lower educational attainment, reduced income, and greater susceptibility to risk-taking behaviors [11]. These consequences may be more pronounced in older adults, who face high-stakes decisions about retirement and long-term planning. Furthermore, this population is a frequent target of financial scams, and higher impulsivity may increase the potential for fraud victimization.

Our results found significant associations between CTB scores and both education and household income, suggesting that both lower socioeconomic (SES) factors and higher pollution exposure are independently associated with greater impulsivity. These findings reinforce the notion that social and environmental factors can be additive in their impacts on behavior. It is possible that these factors may also compound each other, potentially creating feedback loops of disadvantage [20, 25, 39]. We note that our SES findings are consistent with prior work showing a robust relationship between SES and delay discounting behavior [23, 24, 26]. What is startling is that the negative effects of air pollution accounted for additional variance above and beyond SES factors (education and income) in discounting behavior. Future research should examine whether individuals facing both high pollution exposure and socioeconomic hardship are at elevated risk for impulsivity-related outcomes and whether interventions targeting inflammation or environmental exposure could mitigate these effects.

There are several strengths of our study that contribute meaningfully to the existing literature. To our knowledge, this is the first study to examine the relationship between air pollution exposure and reward-based decision making in humans. More broadly, research on the cognitive effects of pollution in the general population remains limited. There is a robust literature showing that prenatal exposure is associated with a range of adverse outcomes in childhood and adolescence [40, 41, 42, 43]; however, studies that examine adult exposure typically focus on clinical disorders such as Alzheimer’s or Parkinson’s disease [2, 44, 45] and thus focus on behaviors relevant to those disorders. While these studies are critical, there is a notable gap in understanding how air pollution may impact the broader adult population.

Our findings help fill this gap by showing that even in otherwise healthy individuals, PM_2.5_ exposure is associated with more impulsive decision-making. It is plausible that similar effects extend to other cognitive domains such as memory and attention given the diffuse nature of inflammation-related damage. Additionally, prior research has shown that in older adults, steeper temporal discounting is associated with poorer episodic memory, so there is potential for significant overlap here [46]. Future work should also examine whether these effects extend to young adults, a population in which the cognitive impacts of pollution remain largely unexplored. This has implications for both individual well-being and population health. Subtle impairments in decision-making and other cognitive abilities may contribute to poorer quality of life and may also serve as early indicators of elevated risk for neurodegenerative diseases. Our results emphasize the importance of studying air pollution’s cognitive impact beyond clinical endpoints.

Another strength of this study lies in the spatial resolution of our pollution exposure estimates. Many US-based studies rely on census-tract level estimates of PM_2.5_, typically derived from the EPA’s network of ground monitors. These monitors are sparse; even in urban centers like Philadelphia, there are only a handful of monitors across the entire metropolitan area [47]. Census-tract estimates assign PM_2.5_ concentrations based on the centroid of each tract and the nearest monitor, which may be several miles away, and often do not account for hyperlocal factors like traffic density, major roadways, or wind patterns [48]. In contrast, we used a high-resolution (1 km x 1 km) model developed by researchers at Washington University of St. Louis, which integrates NASA satellite data, ground monitor readings, meteorological patterns, and emission sources [19]. While such models are increasingly used in environmental science, they remain underutilized in cognitive and health research. Our study highlights the value of interdisciplinary collaboration and underscores the importance of precise spatial modeling for exposure estimation.

Nonetheless, this study has limitations. First, its cross-sectional design precludes any conclusions about causality. It is possible that unmeasured confounding variables explain the observed association between PM_2.5_ exposure and delay discounting. Second, the CTB task was administered using hypothetical rather than incentivized monetary rewards. While prior work suggests that hypothetical and real rewards produce correlated delay discounting estimates, the two are not identical, and incentivized tasks may serve as a better proxy for real-world decision-making [49, 50]. Additionally, our study focused on overall discounting behavior, rather than trial type (e.g., “now vs. later” vs. “later vs. even later”). Research has shown that individuals often display a present bias, or a disproportionate preference for immediate rewards when one option is available “now” even if they would choose the larger, later option when both rewards are shifted into the future [14, 51]. While our sample size limited the feasibility of examining these distinctions, future work should explore whether the relationship between pollution exposure and discounting varies across trial types. Third, while we excluded participants who reported any neurological conditions, our sample may have included participants with undocumented cognitive impairment, which could have influenced results. Additionally, our pollution exposure estimates are based solely on participants’ current addresses. The parent study did not collect residential history, so it is unclear how long participants lived at their reported address. We attempted to mitigate this limitation by calculating a long-term average PM_2.5_ concentration (2012–2022), and annual estimates show that relative exposure levels between neighborhoods have remained fairly stable across this period. That is, areas with the highest PM_2.5_ in 2022 were also among the highest throughout the decade. While we are confident that our results capture relative residential exposure differences, we caution against interpreting them as precise cumulative dose estimates. Furthermore, our estimates of PM_2.5_ exposure do not account for exposure at places of work, leisure, or other locations outside the home. Even if this study were to perfectly capture an individual’s daily mobility and residential history, PM_2.5_ concentration data are estimated over space and time and may not perfectly capture exposure at a particular location and moment. These are ongoing issues in air pollution research. Future longitudinal research and continued innovation on data collection techniques are needed to establish the directionality and temporal dynamics of this relationship.

The need for additional research in this area is urgent. Every year, outdoor air pollution is responsible for approximately 4 million deaths globally [25]. In the USA, this threat is growing, with the concentration of PM_2.5_ increasing 10% from 2016 to 2023, driven by increased urbanization, wildfires, and rising temperatures [52]. As air pollution continues to pose a threat to public health, understanding its effects on cognition will be essential for shaping evidence-based policies and targeted interventions. Our findings underscore the importance of viewing environmental exposures as modifiable risk factors not only for physical health but also for behavioral and cognitive outcomes. Moving forward, interdisciplinary research that integrates environmental science, neuroscience, and public health will be critical for uncovering the mechanisms linking pollution to brain function and identifying populations at greatest risk. By illuminating these connections, we can begin to design strategies that mitigate harm and promote cognitive resilience among people and communities exposed to high levels of air pollution.

## Supplementary information

Below is the link to the electronic supplementary material.

Supplementary file (DOCX 539 KB)

## Data Availability

Portions of the dataset have been released previously and are described in Smith et al. [15]. Additional data will be deposited in the OpenNeuro repository at the conclusion of the study (anticipated 2026–2027). In the meantime, data are available from the corresponding author upon request.
