# Supplementary material for: Air pollution and impulsive choice in aging: evidence from delay discounting
Source: GeroScience. 2025 Sep 24;48(3):4263–73. doi: 10.1007/s11357-025-01906-0 (PMC13356162; doi:10.1007/s11357-025-01906-0)
Supplement: Supplementary file 1 — Supplementary file (DOCX 539 KB) [file 11357_2025_1906_MOESM1_ESM.docx]

Supplement:

Air Pollution and Impulsive Choice in Aging: Evidence from Delay Discounting

*GeroScience*

Maya R. Kilcullen^1^, Jamie-Nicole Luistro^1^, Melanie Kos^1^, Jeremy Mennis^2^, David V. Smith^1^, Ingrid R. Olson^1^

^1^ Department of Psychology and Neuroscience, Temple University, Philadelphia, PA, USA

^2^ Department of Geography, Environment, and Urban Studies, Temple University, Philadelphia, PA, USA

Address correspondence to:

| Maya R. Kilcullen  Department of Psychology and Neuroscience  Temple University  1701 N. 13^th^ Street  Philadelphia, PA 19122 | Email: maya.kilcullen@temple.edu |
| --- | --- |

Keywords: impulsivity, reward, environment, pollution, aging

**SUPPLEMENT**

1. **Convex Time Budget (CTB) Utility Model**

The Convex Time Budget (CTB) task was originally developed by Andreoni and Sprenger (2012) to estimate individual delay discounting parameters using a utility-based modeling approach. Unlike traditional delay discounting tasks that present binary choices between immediate and delayed rewards, the CTB offers a scaled decision format with six possible pairwise monetary allocations per trial, spanning both “now vs. later” and “sooner vs. later” timeframes. This richer structure allows for the estimation of three distinct parameters from participants’ choices:

$$x_{t}=20\cdot\frac{\left( \beta^{t_{0}}\delta^{k}P \right)^{\frac{1}{\alpha-1}}}{1+P\left( \beta^{t_{0}}\delta^{k}P \right)^{\frac{1}{\alpha-1}}}$$

where:

- α captures the curvature of the utility function, reflecting sensitivity to reward magnitude,
- δ reflects the long-term discounting rate (i.e., patience),
- β captures present bias, or the added weight given to immediate rewards (when the sooner option is "today"),
- t0​ is a binary indicator for immediacy (1 if “now”, 0 otherwise),
- k is the time difference between sooner and later rewards,
- P reflects the implied interest rate between the options.

We first estimated individual-level parameters using non-linear least squares (NLS), followed by a non-linear mixed effects (NLME) model that allowed the delta (δ) parameter to vary by participant. This analysis was done using an R script provided by Rita Ludwig and Michael Kuhn at the University of Oregon, designed in accordance with the utility model proposed by Andreoni & Sprenger [1-3]. Due to convergence issues when estimating all three parameters with participant-level random effects, we focused our analysis on the delta (δ) parameter as the most stable and interpretable index of delay discounting under these conditions.

Estimated delta (δ) scores in our sample ranged from 0.982 to 1.002 (M = 0.998, SD = 0.0066). We then conducted a linear regression analysis using δ as the dependent variable and long-term PM_2.5_ exposure as the independent variable, controlling for age, gender, education, and household income. Results indicated that greater PM_2.5_ exposure was significantly associated with lower delta (δ) values (adjusted β = -0.20, p = 0.018), indicating steeper discounting.

Importantly, delta (δ) was highly correlated with the average CTB score (r = 0.94), which ranged from 1 to 6 (M = 3.88, SD = 1.67). Given the greater variability and ease of interpretation of average CTB scores, our primary analysis focused on that summary metric, with this modeling analysis offered in support.

**Supplementary Fig 1** Linear Regression Model using CTB delta (δ)


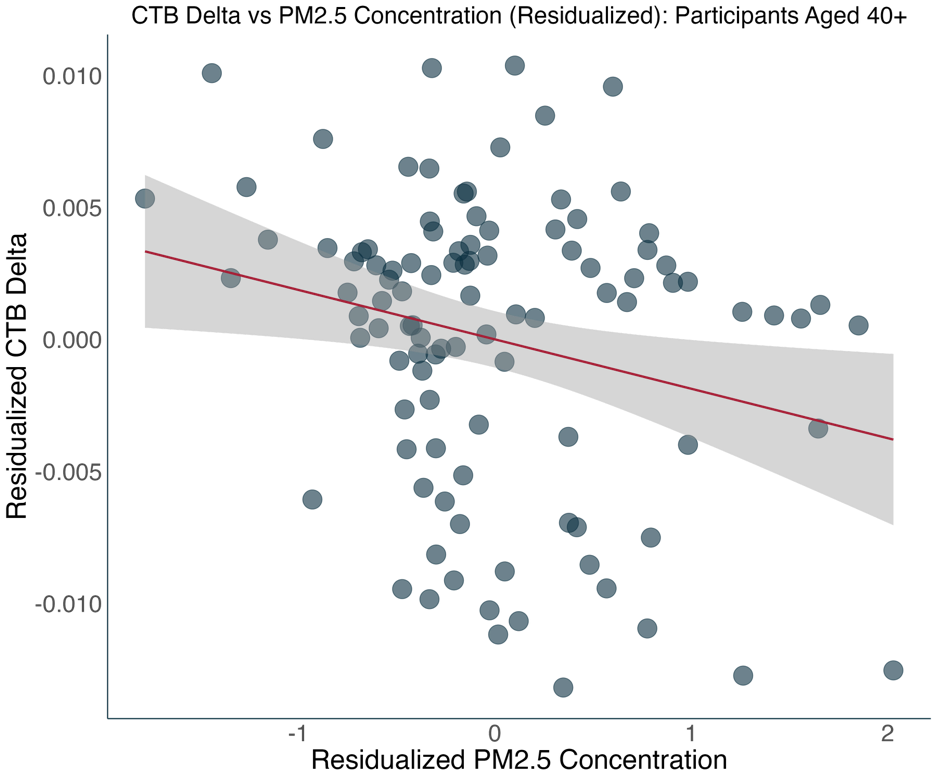


**Supplementary Table 1** GLM Statistics using CTB delta (δ) as the dependent variable

| **Predictor** | **B (Unstd.)** | **SE** | **β (Std.)** | **t** | **p** |
| --- | --- | --- | --- | --- | --- |
| (Intercept) | 1.01 | 7.91 x 10^-3^ | – | 126.977 | <0.001 |
| PM_2.5_ Concentration | -1.87 x 10^-3^ | 7.76 x 10^-4^ | -0.18 | -2.411 | 0.018 * |
| Age | -4.36 x 10^5^ | 5.20 x 10^-5^ | -0.06 | -0.837 | 0.405 |
| Gender (1 = Female) | 1.46 x 10^3^ | 1.16 x 10^-3^ | 0.12 | 1.261 | 0.210 |
| Education (Scale) | 1.39 x 10^-3^ | 3.71 x 10^-4^ | 0.36 | 3.736 | <0.001*** |
| Household Income (Scale) | 7.76 x 10^-4^ | 3.51 x 10^-4^ | 0.26 | 2.211 | 0.029 ** |

**Model Summary:**

*R^2^ = 0.325, Adjusted R^2^ = 0.291, F(5, 97) = 9.36, P < 0.001; Residual SE = 0.006*

1. **Correlation Matrix**

Prior to regression modeling, we ran a correlation matrix with demographic variables of interest: Age, Gender, Education, Household Income, Personal Income, Social Ladder. The Social Ladder variable is a subjective, self-reported measure in which participants rated where they viewed themselves on a social ladder (1-10; 10 representing people who are the “best off”). Following this analysis, Social Ladder and Personal Income were excluded from the regression model due to strong correlation with household income and education.


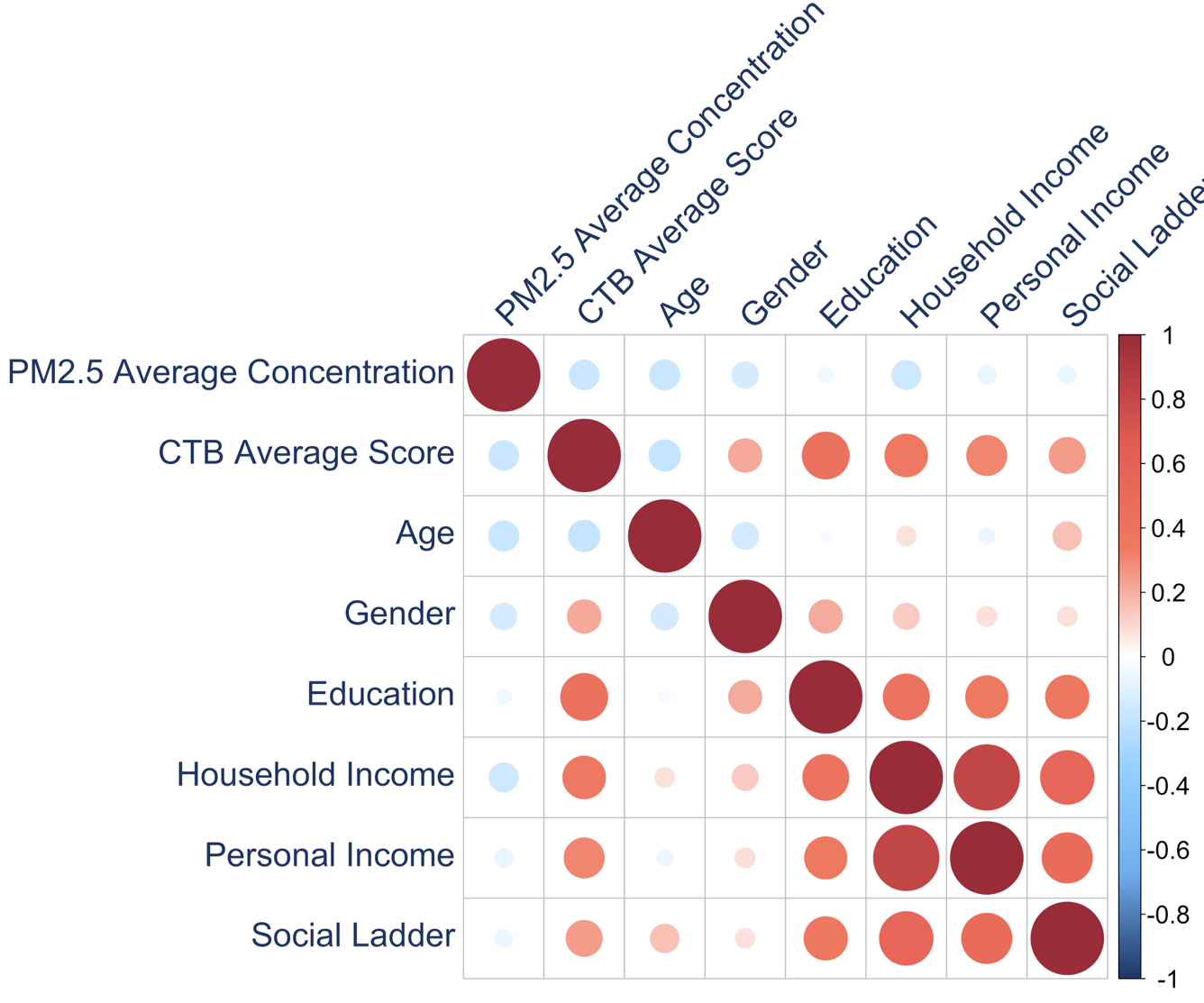
**Supplementary Fig 2** Demographic Variable Correlation Matrix

**References**

1. Andreoni, J., Kuhn, M. A., & Sprenger, C. (2015). Measuring time preferences: A comparison of experimental methods. *Journal of Economic Behavior & Organization*, *116*, 451–464. <https://doi.org/10.1016/j.jebo.2015.05.018>
2. Kuhn, M. A. (n.d.). *Time Preference Estimation Kit*. Michael A. Kuhn. Retrieved June 26, 2025, from <https://www.makuhn.net/time-preference-estimation-kit>
3. Ludwig, R. M., Flournoy, J. C., & Berkman, E. T. (2019). Inequality in personality and temporal discounting across socioeconomic status? Assessing the evidence. *Journal of Research in Personality*, *81*, 79–87. <https://doi.org/10.1016/j.jrp.2019.05.003>
